# Therapeutic Effects of Salvianolic Acid B on Angiotensin II–Induced Atrial Fibrosis by Regulating Atrium Metabolism via Targeting AMPK/FoxO1/miR-148a-3p Axis

**DOI:** 10.1007/s12265-022-10303-3

**Published:** 2022-08-19

**Authors:** Jie Liu, Qijuan Sun, Xiaotong Sun, Qian Wang, Guangchen Zou, Dewei Wang, Baoxiang Zhuang, Zhaodong Juan, Rui Zhang, Daoliang Zhang

**Affiliations:** 1 Shandong Provincial Medicine and Health Key Laboratory of Clinical Anesthesia, School of Anesthesiology, Weifang Medical University, Weifang, China; 2 Department of Cardiology, XinHua Hospital Affiliated to Shanghai Jiao Tong University School of Medicine, Shanghai, China; 3 Danbury Hospital, Danbury, CT USA; 4 Department of Cardiology, Shanghai Chest Hospital, Shanghai Jiaotong University, Shanghai, China; 5 Department of Cardiology, Fuwai Hospital Chinese Academy of Medical Sciences, Shenzhen, China

**Keywords:** Atrial fibrosis, Salvianolic acid B, Metabonomics, AMPK, FoxO1

## Abstract

The present study highlights the effects of salvianolic acid B (Sal B) on angiotensin II (Ang II)–activated atrial fibroblasts as well as the associated potential mechanism from the metabonomics perspective. Metabolic profile analysis performed an optimal separation of the Ang II and control group, indicating a recovery impact of Sal B on Ang II–activated fibroblasts (FBs). We found that metabolite levels in the Ang II + Sal B group were reversed to normal. Moreover, 23 significant metabolites were identified. Metabolic network analysis indicated that these metabolites participated in purine metabolism and FoxO signaling pathway. We found that Sal B activated AMP-activated protein kinase (AMPK) phosphorylation, which further promoted FoxO1 activation and increased miR-148a-3p level. We further verified that Sal B modulate the abnormal AMP, phosphocreatine, glutathione (GSH), and reactive oxygen species (ROS) production in Ang II–stimulated FBs. Collectively, Sal B can protect the Ang II–activated FBs from fibrosis and oxidative stress via AMPK/FoxO1/miRNA-148a-3p axis.

**Graphical abstract:**

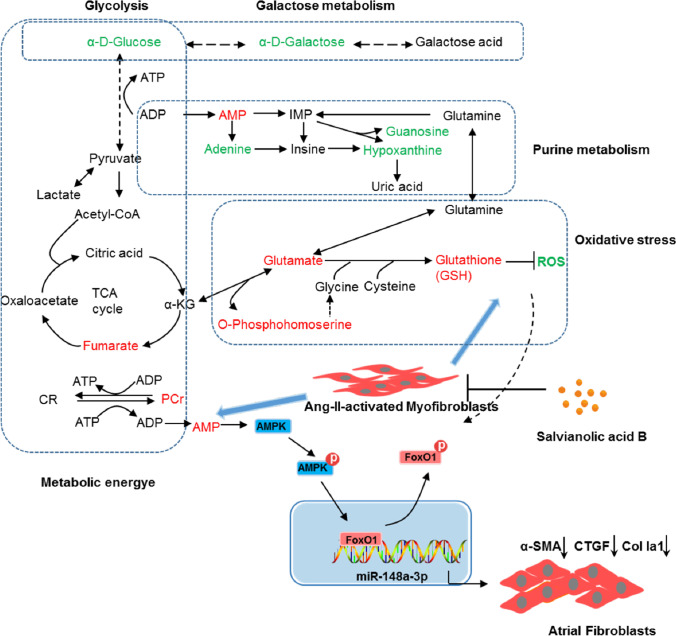

## Introduction

Atrial fibrillation (AF) is a common arrhythmia and associated with an increased risk of heart failure, stroke, and mortality [1]. The mechanism of AF is not fully understood but includes structural remodeling, electrical remodeling, autonomic nerve system remodeling, and Ca^2+^-handling abnormalities. Among abovementioned pathophysiological mechanism, structural remodeling represented by atrial fibrosis is of vital importance. Despite this understanding, currently available therapeutic approaches have limited efficacy, and the occurrence and refractory of AF are often accompanied by more atrial fibrosis.

Atrial fibrosis is characterized by the accumulation of extracellular matrix (ECM) proteins in the interstitium of atrium tissue, including collagen I (Col1a1), collagen III (Col3a1), fibronectin, and elastin [2]. As the most prevalent constituent cell type in the heart, cardiac fibroblasts (FBs) play a key role in the pathogenesis of atrial fibrosis. In the diseased state of atrial fibrosis, activated FBs could differentiate to more active myofibroblasts (MFBs). MFBs highly expressed alpha smooth actin (α-SMA), resulting in the deposition of ECM. Excessive atrial fibrosis impaired the electromechanical coupling of atrial cardiomyocytes and then facilitated the arrhythmogenesis of AF [3]. Hence, activated FBs and MFBs were regarded as the therapeutic target of atrial fibrosis and potentially as the therapeutic target for AF.

Salvianolic acid B (Sal B), a hydrophilic phenolic acid, is one of the most abundant and biologically active ingredients extracted from the plant *Salvia miltiorrhiza* Radix [4]. Sal B has been reported to have antioxidant, anti-inflammatory, anti-apoptosis, and anti-fibrotic effects [5]. Besides, Sal B showed protective effects in multiple organs, including cardiovascular and cerebrovascular diseases [6, 7], liver or pulmonary fibrosis [4, 8], aging, and acute renal injury [9]. Recently, Wang et al. [10] found that Sal B could alleviate angiotensin II (Ang II)–induced cardiac fibrosis via NF-κB pathway *in vitro*. Although multiple traditional molecular biology studies have provided mechanistic insights into the effects of Sal B, a comprehensive study using metabolomic, a systems biology method, has never been done before. Metabolomics involves quantitatively assessing all the metabolites in a biological system in order to paint an overview of the different metabolic pathways [11]. Metabolomics is a useful tool for precision medicine, which comprehensively profile cardiovascular disease pathogenesis and offer new cardiovascular disease biomarkers. Combining metabolic changes with previous biological knowledge, we can quickly uncover mechanisms related to the pathogenesis of diseases or the pharmacology of drugs [12]. Over the past few decades, metabolomics has significantly contributed to the discovery of the pathophysiology and biomarkers of cardiovascular diseases [13–15]. Cultured primary cells have been widely used in both basic research and secondary compound profiling, notably *in vitro* studies assessing drug metabolism and toxicity [16, 17].

Based on the above research background, we studied the effects of Sal B on atrial fibrosis using Ang II–activated atrial fibroblasts as a cell model, via utilizing metabolomics analysis to further clarify its underlying mechanisms.

Previous metabolomic studies have suggested a protective effect of Sal B in different disease models. Sal B has been shown to ameliorate ischemia-induced injury in a stroke model through improved energy metabolism[18], lipid metabolism, inflammatory responses [19], and oxidant stress [20]. A similar protective effect has been shown for Sal B in a model of myocardial ischemia [21]. However, the changed metabolites in response to the Sal B therapy in atrial fibrosis have never been reported before. In this study, metabolomic analysis was used to evaluate the therapeutic effects of Sal B on atrial fibrosis. After Sal B preconditioning, metabolomic analysis of Ang II–activated FBs was performed to elucidate the underlying mechanism of Sal B on atrial fibrosis. Multivariate analysis, including principle component analysis (PCA), partial least squares-discriminate analysis (PLS-DA), and orthogonal partial least squares-discriminate analysis (OPLS-DA) score plots, was used to identify the metabolites involved in the anti-fibrotic mechanism of Sal B. Finally, biological events associated with these metabolites were verified using conventional methods in molecular biology *in vitro*.

## Materials and Methods

### Ethics Statement

All animal experiments conformed to the Guide for the Care and Use of Laboratory Animals published by Weifang Medical University and were approved by the Committee for Experimental Animal Ethics of Weifang Medical University (Approval No. 2021SDL059).

### Animals and Treatments

Eight-week-old male BALB/c mice (body weight 22–25 g) were purchased from Pengyue Laboratory Animal Co., Ltd. (Shandong, China). All mice were housed in a room under controlled temperature and humidity conditions, with a 12-h light-12-h dark cycle, and had free access to food and water. The mice were acclimatized to the laboratory environment for 7 days before the studies. For in vivo study, all animals were anesthetized with 2% isoflurane inhalation before euthanization. Twenty-four mice were randomized into three groups, namely the sham procedure group, Ang II treatment group, and Ang II and Sal B co-treatment (Ang II + Sal B) group. An osmotic pump (ALZET 2004, Cupertino, CA, US) filled with Ang II (1.0 mg/kg/min) (CAS:4474–91-3, #A9290, Solarbio, Beijing, China) or PBS (200 μl) was implanted subcutaneously after anesthesia. In the Ang II + Sal B group, Sal B (CAS: 121521–90-2, #S123658, Jingchun Biochemical Technology, Shanghai, China) was given by tail veins of 200 mg/kg once daily for 4 weeks after establishing the experimental animal model [10].

### Isolation of FBs and Differentiation of Fibroblasts into MFBs

Both atria were cut off from the hearts of suckling 1- to 3-day-old neonatal BALB/c mice. The atrium tissues were cut into 1mm^3^ with ophthalmic scissors and then were digested with 0.1% collagenase II (#LS004174, Worthington, USA) stirred with magnetic stir (60 rpm/min) at 37 °C for 30 min. After filtered and centrifugated at 1000 rpm/min for 5 min, FBs were resuspended and cultured in Dulbecco’s Modified Eagle’s Medium (DMEM, 11,995, Solarbio, Beijing, China) containing 10% fetal calf serum (FBS, #10099141C, Gibco, USA) and 100 U/L penicillin/streptomycin (#P1400, Solarbio, Beijing, China) for 1.0 h. Then fresh medium was added to the plate. FBs in passages 2–3 were used for the following study.

To promote the differentiation of FBs into MFBs, culture media was replaced by DMEM containing Ang II (100 nM) for 24 h when cell fusion has reached to 60% [22]. Immunofluorescence staining for α-SMA was used to confirm successful activation and trans-differentiation of FBs into MFBs.

### Cell Viability Assay

Cell counting kit 8 (CCK8, #CA1210, Solarbio, Beijing, China), which is a detection for cell viability, was performed according to the manufacturer’s protocols, as described previously with minor modifications [23]. The FBs (5 × 10^3^ cells/well) were plated in 96-well plates for 24 h, and then the cells were incubated with serum-free medium for 6 h. After being pretreated with 12.5, 25.0, and 50.0 μM Sal B for 2 h [24], FBs were stimulated by 100 nM Ang II. After Ang II incubation for 24 h, 10 μl CCK8 was added to each well in plate, and incubated for a further 4 h. The absorbance was detected at 450 nm using a microplate reader.

### 5-Ethynyl-20-Deoxyuridine Assay

Proliferation of FBs was investigated with BeyoClickTM EdU Cell Proliferation Kit (#C0075S, Beyotime Biotechnology, Shanghai, China) according to the manufacturer’s protocols, as previously reported [25]. In brief, The FBs were washed with PBS twice and incubated with 5-ethynyl-20-deoxyuridine (EdU) working solution (10 µM) for 4 h at 37 °C in the dark. After incubation, FBs were washed with PBS twice and fixed with 4% paraformaldehyde (#P1110, Solarbio, Beijing, China) at room temperature for 15 min. Next, the cells were permeabilized with 0.1% Triton X100 for 15 min and washed with PBS three times. Then cells were incubated with Click reaction solution for 30 min at 37 °C in the dark. Finally, the cells were incubated with 4,6-diamidino-2-phenylindole (DAPI, #C0060, Solarbio, Beijing, China) in the dark for 5 min. Images were captured with fluorescence microscope (BX-53, Olympus Corporation, Tokyo, Japan). FBs that undergo DNA replication during incubation present red fluorescence while the nucleus was represented with blue fluorescence.

### Measurement of Glutathione and Reactive Oxygen Species Detection

For the analysis, after being pretreated with 12.5, 25.0, and 50.0 μM Sal B for 2 h, FBs were stimulated by 100 nM Ang II for 24 h. The cell supernatants were then collected for the analysis. The level of glutathione (GSH) was quantified by using the GSH ELISA kit (**#**E-EL-0026c, Elabscience, Wuhan, China), in accordance with the manufacturer’s instructions. The reactive oxygen species (ROS) level was examined in cells using 2′,7′-dichlorofluorescin diacetate for 30 min at 37 °C. The cells were observed, and data were analyzed through microplate reader.

### Histology

The hearts were quickly removed under deep anesthesia with pentobarbital sodium (60 mg/kg, i.p.). The left atrium was fixed with 4% paraformaldehyde at 4 °C overnight and dehydration with gradient alcohol. The tissues were embedded in paraffin and cut into 5-µm-thick sections. The sections were stained with Masson’s trichrome staining (#G1340, Solarbio, Beijing, China) to assess the degree of atrial fibrosis. Meanwhile, 5-μm-thick sections were stained with hematoxylin and eosin (H&E) (#G1120, Solarbio, Beijing, China) to evaluate the morphological changes of atrial muscle tissues. Finally, the sections were viewed and photographed under a microscope and the images were analyzed by Image-Pro Plus 6.0 software.

### Immunofluorescence Staining

The cells were seeded on poly-lysine-coated glass slides. The cells were fixed with 4% paraformaldehyde according to the manufacturer’s instructions, washed three times with PBS and blocked with goat serum (#SL038, Solarbio, Beijing, China) for 30 min at room temperature. And for heart tissue immunofluorescence staining, the 5-μm-thick sections were only blocked with goat serum for 1 h at room temperature. Then the primary antibody was then added and incubated overnight at 4 °C. Secondary antibody was added after the cells were washed with PBS for three times and incubated for 40 min at 37 °C in the dark. Next, the nuclei were stained with DAPI in the dark for 5 min. Images were then taken with fluorescence microscope.

### Quantitative Polymerase Chain Reaction

Total RNA from cells was isolated using RNeasy Mini Kit (#74,104, Qiagen, NV, Netherlands) according to the manufacturer’s instructions and determined by Naonodrop OneC Microvolume UV–Vis Spectrophotometer (Thermo Fisher Scientific, Waltham, MA, USA). To measure the mRNA expression, total RNA was reverse transcribed to complementary DNA using the Hiscript II Q RT SuperMix for qPCR (+ gDNA wiper) (#R223-01, Vazyme, Nanjing, China) and Bulge-Loop miRNA quantitative polymerase chain reaction (qRT-PCR) Starter Kit (#C10211-2, RiboBio, Guangzhou, China). qRT-PCR reactions were performed with ABI 7500 Real-Time PCR System (Applied Biosystems, CA, USA) using ChamQ Universal SYBR qPCR Master Mix (#Q711-02, Vazyme, Nanjing, China) and Bulge-Loop miRNA qRT-PCR Starter Kit (#C10211-2, RiboBio, Guangzhou, China). The relative mRNA expression index was normalized with GAPDH or U6 expression levels using the 2^−ΔΔCt^ method. Primer sequences are listed in Table 1.

**Table 1:** List of primers used for quantitative real-time PCR

Gene	Forward primer	Reverse primer
Mouse-FoxO1	TCGAACCAGCTCAAACGC	GGTGGATACACCAGGGAATG
Mouse-AMPK	GCTCGCAGTGGCTTATCAT	TGGACAGCGTGCTTTGG
Mouse-miR-148a-3p	AGCAGTTCAGTGCACTACAG	GCAGGGTCCGAGGTATTC
Mouse-GAPDH	GTTACCAGGGCTGCCTTCTC	ACCAGCTTCCCATTCTCAGC

### Western Blot Analysis

Western blot analysis was performed as described previously with minor modifications [26]. The cells were lysed with RIPA lysis buffer (#P0013B, Beyotime Biotechnology, Shanghai, China) containing 1% protease and phospholipase inhibitor on ice for 30 min and centrifuged at 12,000 rpm in a cold centrifuge at 4° C for 10 min. The protein concentration of each sample was quantified using BCA kit (#PC0020, Solarbio, Beijing, China). Then the protein samples were electrophoresed on an 8–10% SDS polyacrylamide gel and transferred to a nitrocellulose membrane (Millipore, MA, USA). Blotting was blocked with 5% skim milk for 3 h, and then incubated overnight at 4 °C with the following primary antibodies: rabbit anti-α-SMA (#19,245,1:1000, CST), rabbit anti-collagen I (#91,144, 1:500, CST), rabbit anti-CTGF (#86,641,1:500, CST), rabbit anti-phospho(p)-AMPK (#2535,1;1000 CST), rabbit anti-AMPK (#2532, 1;1000, CST), rabbit anti-phospho(p)-FoxO1 (#9461, 1;1000, CST), rabbit anti-FoxO1 (#2880, 1;1000, CST), and rabbit anti-GAPDH (#5174, 1:1000,CST). Then, the membrane was washed three times with PBS, and incubated with horseradish peroxidase–conjugated secondary antibody (Goat anti-Rabbit, A0208, 1:7000, Beyotime Biotechnology, Shanghai, China) for 1 h at room temperature. The membrane was washed with PBS for three times again, and exposed to enhanced chemiluminescence detection reagents (#CW0049, CWBIO, Beijing, China) and quantified using Image J software.

### Sample Preparation of Metabolic Study

Sample preparation was carried out according to the method as previously reported by Chen et al. [27]. The 150-μL aliquot of methanol (#R40121, Thermo Fisher Scientific, Waltham, MA, USA) (containing 100 ng/mL l-2-chlorophenylalanine (CAS: 103616–89-3, Hengchuang Bio-technology, Shanghai, China), in positive ion mode or 1 μg/mL ketoprofen in negative ion mode as the internal standard), was added to 100 μL cell precipitate in a 1.5-mL tube. The mixture was vortexed for 30 s, and then centrifuged at 13,000 rpm/min for 10 min at 4 °C; 200 μL of the supernatant was transferred into another 1.5-mL tube. The supernatant dried under a gentle stream of nitrogen at room temperature, redissolved with 200 μL methanol solution, vortexed for 30 s, and centrifuged at 13,000 rpm/min for 10 min at 4 °C. A total of 150 μL of the supernatant was transferred into vials. Finally, the 1-μL aliquot of the supernatant fraction was injected into ultra-performance liquid chromatography/quadrupole time-of-flight mass spectrometry (UPLC-Q/TOF–MS) for analysis.

### UPLC-Q/TOF–MS Analysis

A Nexera UPLC system (Shimadzu Corporation, Japan) coupled with Q-Exactive quadrupole Orbitrap mass spectrometer equipped with heated electrospray ionization (ESI) source (Thermo Fisher Scientific, Waltham, MA, USA) was used to analyze the metabolic profiling in both ESI positive and ESI negative ion modes. An ACQUITY UPLC HSS T3 column (1.8 μm, 2.1 × 100 mm) was employed in both positive and negative modes. The binary gradient elution system consisted of (A) pure water (containing 0.1% formic acid, v/v) (#85,170, Thermo Fisher Scientific, Waltham, MA, USA) and (B) acetonitrile (containing 0.1% formic acid, v/v) (#85,174, Thermo Fisher Scientific, Waltham, MA, USA). Separation was achieved using the following gradient: 0 min, 5% B; 2 min, 5% B; 4 min, 25% B; 8 min, 50% B; 10 min, 80% B; 14 min, 100% B; 15 min, 100% B; 15.1 min, 5% and 16 min, 5% B. The flow rate was 0.35 mL/min and the column temperature was 45 °C. All the samples were kept at 4 °C during the analysis. The injection volume was 2 μL. The mass range was from m/z 125 to 1000. The resolution was set at 70,000 for the full MS scans and 17,500 for HCD MS/MS scans. The collision energy was set at 10, 20, and 40 eV. The mass spectrometer operated as follows: spray voltage, 3500 V ( +) and 3500 V ( −); sheath gas flow rate, 40 arbitrary units ( +) and 35 arbitrary units ( −); auxiliary gas flow rate, 10 arbitrary units ( +) and 8 arbitrary units ( −); capillary temperature, 32 °C.

The QCs were injected at regular intervals (every 6 samples) throughout the analytical run to provide a set of data from which repeatability can be assessed.

### Statistical Analysis

The metabonomic analysis was done by Oebiotech (Shanghai, China). The original LC–MS data were processed by software Progenesis QIV2.3 (Nonlinear, Dynamics, Newcastle, UK) for baseline filtering, peak identification, integral, retention time correction, peak alignment, and normalization. Main parameters of 5-ppm precursor tolerance, 10-ppm product tolerance, and 5% product ion threshold were applied. Compound identification was based on precise mass-to-charge ratio (M/Z), secondary fragments, and isotopic distribution using the Human Metabolome Database (HMDB), Lipidmaps (V2.3), Metlin, EMDB, PMDB, and self-built databases to carry out the qualitative analysis.

The extracted data were then further processed by removing any peaks with a missing value (ion intensity = 0) in more than 50% in groups, by replacing 0 value by half of the minimum value, and by screening according to the qualitative results of the compound. Compounds with resulting scores below 36 (out of 60) points were also deemed to be inaccurate and removed. A data matrix was combined from the positive and negative ion data.

The matrix was imported in R to carry out PCA to observe the overall distribution among the samples and the stability of the whole analysis process. OPLS-DA and PLS-DA were utilized to distinguish the metabolites that differ between groups. To prevent overfitting, sevenfold cross-validation and 200 response permutation testing were used to evaluate the quality of the model.

Variable importance of projection (VIP) values obtained from the OPLS-DA model were used to rank the overall contribution of each variable to group discrimination. A two-tailed Student’s *T*-test was further used to verify whether the metabolites of difference between groups were significant. Differential metabolites were selected with VIP values greater than 1.0 and *p*-values less than 0.05.

The other statistical analyses were performed using GraphPad Prism 6. The results were expressed as mean ± standard deviation. Differences between groups were compared with one-way ANOVA or *t*-tests as appropriate. *p*-value less than 0.05 presented statistical significance. The neurological deficit score was expressed as the median and analyzed using Kruskal–Wallis test and differences between groups were compared with Mann–Whitney *U* test.

## Results

### Sal B Attenuated Ang II–Induced Atrial Fibrosis

**Fig. 1:**
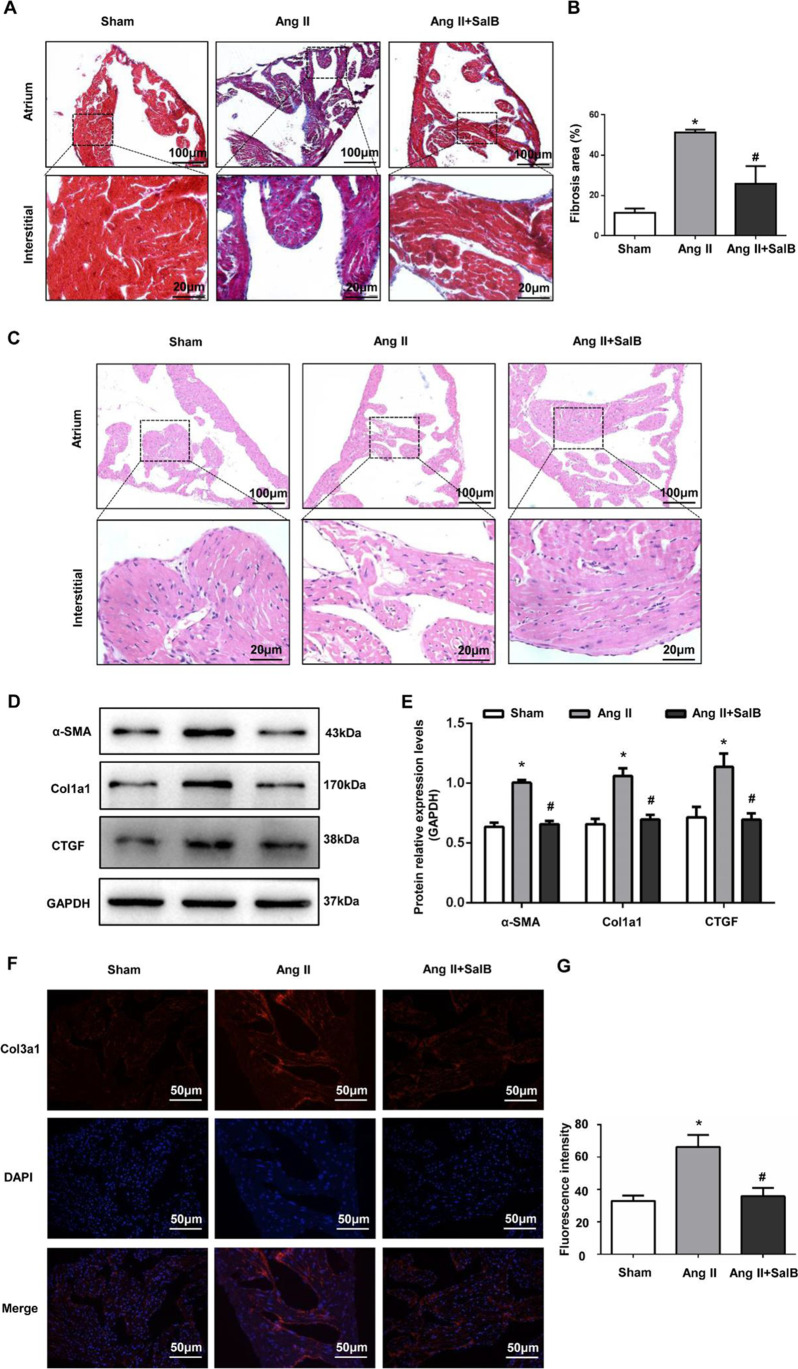
Sal B attenuated Ang II–induced atrial fibrosis. **A** Masson’s trichrome staining of hearts from sham and Ang II–treated mice and Ang II + Sal B–treated mice. Scale bar = 100 μm and 20 μm. **B** Quantification of interstitial fibrosis by calculating collagen volume fraction 4 weeks after operation. **C** HE staining of hearts from sham and Ang II–treated mice and Ang II + Sal B–treated mice. Scale bar = 100 μm and 20 μm. **D**, **E** Western blot analysis showed the protein expression levels of α-SMA, Col1a1, and CTGF in atria tissues from sham and Ang II–treated mice and Ang II + Sal B–treated mice. **F**, **G** Immunofluorescence staining analysis showed the protein expression levels of Col3a1 in atria tissues from sham and Ang II–treated mice and Ang II + Sal B–treated mice. Scale bar = 50 μm. Data were presented as mean ± SEM (*n* = 8 in each group). *p < 0.05 vs Sham group; #*p* < 0.05 vs Ang II group

Masson’s trichrome staining was used to determine the degree of atrial fibrosis. The results showed that Ang II significantly increased the degree of fibrosis in the interstitium and perivascular areas (collagen, blue color), compared with the Sham group. However, atrial fibrosis was significantly less pronounced in the Ang II + Sal B group compared with the Ang II group (Fig. 1A, B). HE staining results showed that pathophysiology of atrial tissues of the Ang II group changed significantly compared with the Sham group. The myocardial cells in the Sham group arranged neatly, but the myocardial cells in the Ang II group were disordered, and there was more focal fibrosis between the tissues. The fibrosis in the myocardial tissue of the Ang II + Sal B group significantly reduced (Fig. 1C). Fibrosis-related protein expression was assessed using Western blot and immunofluorescence staining. The expressions of α-SMA, Col1a1, CTGF, and Col3a1 were significantly higher in the Ang II group compared with those in the Sham group while their expressions were significantly lower in the Ang II + Sal B group compared with the Ang II group (Fig. 1D–G).

### Effects of Sal B on the Cell Viability, Proliferation, Secretion Functions, and Oxidative Stress of MFBs

**Fig. 2:**
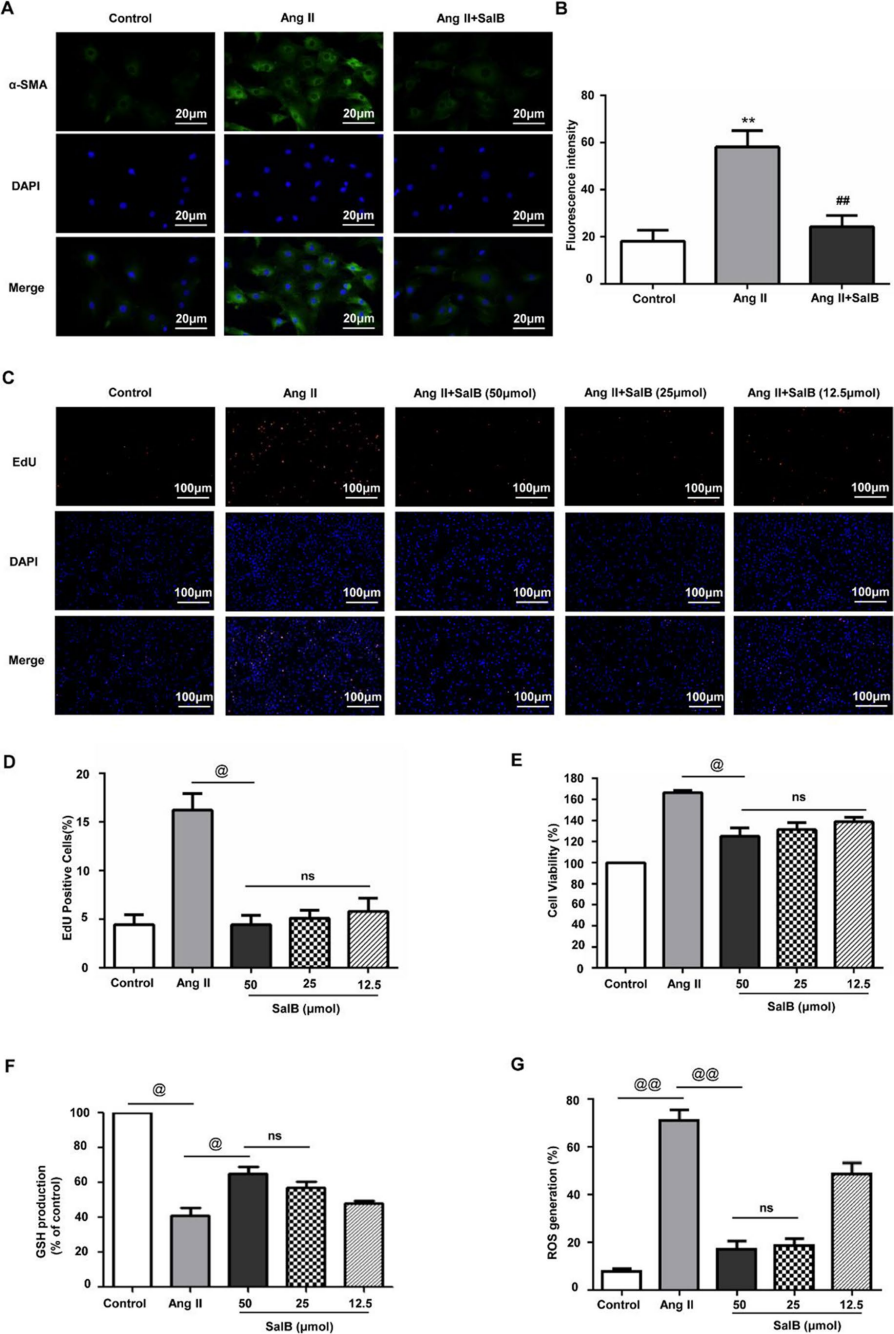
Effects of Sal B on the proliferation, secretion functions, and oxidative stress of MFBs. **A** Untreated FBs (control), Ang II–treated MFBs, and Ang II + Sal B–treated MFBs were stained with α-SMA (green) and DAPI (blue). Scale bar = 20 μm. **B** The fluorescence signals of different groups were analyzed using ImageJ. **C**, **D** Representative fluorescence images of EdU staining of FBs. Proliferating FBs positively stained with EdU showed red color. Cell nuclei stained with DAPI showed blue color. Scale bar = 100 μm. **E** Cell viability treated with PBS (control), Ang II, or dedicated concentrations of Sal B (12.5 μg/ml, 25 μg/ml, or 50 μg/ml). **F** ELISA assay showed the level of GSH in the control group, Ang II group, and Ang II + Sal B group. **G** ROS level was examined in the control group, Ang II group, and Ang II + Sal B group. Data were presented as mean ± SEM (*n* = 3 in each group). *p < 0.05 vs control group, **p < 0.01 vs control group; #*p* < 0.05 vs Ang II group, ##*p* < 0.01 vs Ang II group; @*p* < 0.05, @@*p* < 0.01; ns, no significance

Excessive production of ECM by FBs induced by the presence of Ang II is an important mechanism for atrial fibrosis. To create a model of atrial fibrosis, Ang II was used to stimulate atrial FBs to trans-differentiate into MFBs. Immunofluorescence staining for α-SMA, a specific marker for MFBs, was used to confirm success of trans-differentiation. Limited amounts of α-SMA were expressed by the untreated FBs while treatment with Ang II significantly upregulated its expression. This upregulation was significantly negated with Sal B treatment, suggesting Sal B can inhibit the upregulation of α-SMA expression by Ang II (Fig. 2A, B). We also studied the cell viability and proliferation of atrial FBs which are an important part of the atrial fibrosis process. After being exposed to different concentrations of Sal B (12.5, 25.0, and 50.0 μM) for 2 h, FBs were stimulated with100 nM Ang II for 24 h. CCK-8 assay was used to assess the viability of the FBs and EdU assay was used to assess the proliferation of the FBs. We found that Ang II could promote the cell viability and proliferation of atrial FBs, but this can be inhibited by pre-treatment with different concentrations of Sal B (Fig. 2C–E). FBs treated with different concentrations of Sal B showed no significant difference in viability and proliferation.

Given that oxidative stress stimulation was a typical characteristic of cardiac fibrosis and remodeling [22], to explore whether Sal B suppresses the activation of FBs induced by Ang II via modulation intracellular oxidative stress, the effects of Sal B on the level of GSH and ROS production were studied in Ang II–stimulated FBs. FBs were stimulated with Ang II, which reduced the level of GSH. However, Ang II–induced decrease of GSH was blunted after pretreatment with Sal B (Fig. 2F). Furthermore, we observed a significant increase level of ROS in FBs treated with Ang II, but Ang II–induced increased ROS production was abolished by pretreatment with Sal B (Fig. 2G). A Sal B concentration of 25.0 μM was used for subsequent experiments.

### Metabolomic Profiling and Multivariate Data Analysis

**Fig. 3:**
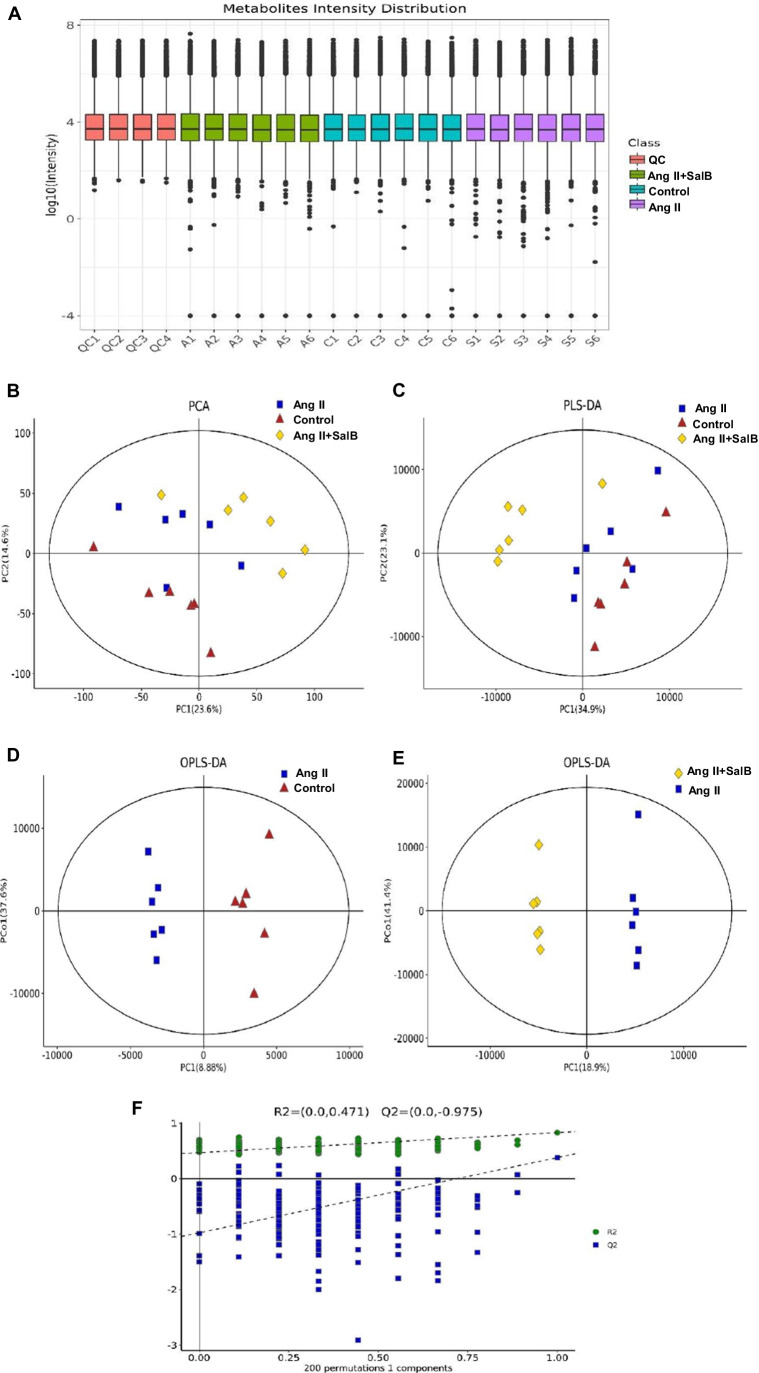
Multivariate statistical analysis of the cell metabolites in Ang II–activated FBs. **A** Distribution of intensities for metabolites in control, Ang II, and Ang II + Sal B. **B** Principal component analysis (PCA) score plot of the control (blue squares), Ang II (red triangles), and Ang II + Sal B (yellow rhombus) groups. **C** PLS-DA score plot of the control (blue squares), Ang II (red triangles), and Ang II + Sal B (yellow rhombus) groups. **D** OPLS-DA models of control and Ang II. The blue squares and red triangles indicated control and Ang II groups, respectively. **E** OPLS-DA models of Ang II + Sal B and Ang II. The red triangles and yellow rhombus indicated Ang II and Ang II + Sal B groups, respectively. **F** Cross-validated score plots of the OPLSDA model (*R*^2^ = 0.816; *Q*.^2^ = 0.972)

The UPLC-Q/TOF–MS system was used to obtain metabolic profiling of the samples in the positive and negative ion modes. Figure 3A showed the typical base peak intensity chromatograms of samples from the control group, Ang II group, and Ang II + Sal B group. To determine whether Sal B would alter the metabolic pattern of Ang II–activated FBs and to determine whether the metabolites concentration would change significantly (namely potential biomarkers), the multivariate data analysis was done, including PCA, PLS-DA, and OPLS-DA based on the UPLCQ/TOF–MS data. In this study, the score plots of the PCA of the control group, Ang II group, and Ang II + Sal B group were clearly distinct, with the Ang II + Sal B group being much closer to the control group than the Ang II group (Fig. 3B). This suggested that Ang II activation changed the metabolic profile of FBs and this change can be partly prevented with Sal B treatment. Additionally, PLS-DA and OPLS-DA were further used to maximize differences between groups (Fig. 3C–E). OPLS-DA models (control vs Ang II; Ang II + Sal B vs Ang II) yielded good classification under different conditions (*R*^2^ = 0.967, *Q*^2^ = 0.508; *R*^2^ = 0.98, *Q*^2^ = 0.75). The response of the permutation test with 200 permutations was used to construct and validate the OPLS-DA model. *R*^2^ and *Q*^2^ values (*R*^2^*X* = 0.816, *Q*^2^*Y* = 0.972) of OPLS-DA model were lower than the original values, which were considered to have good predictive power and reliability (Fig. 3F). These results confirmed changes in the metabolic profiles of FBs induced by Ang II and the protective effect against such effects by Sal B. The OPLS-DA was built to clarify distinctions and potential biomarkers among groups.

### Sal B Ameliorates the Perturbations in Purine Metabolism and FoxO Signaling Pathway

**Fig. 4:**
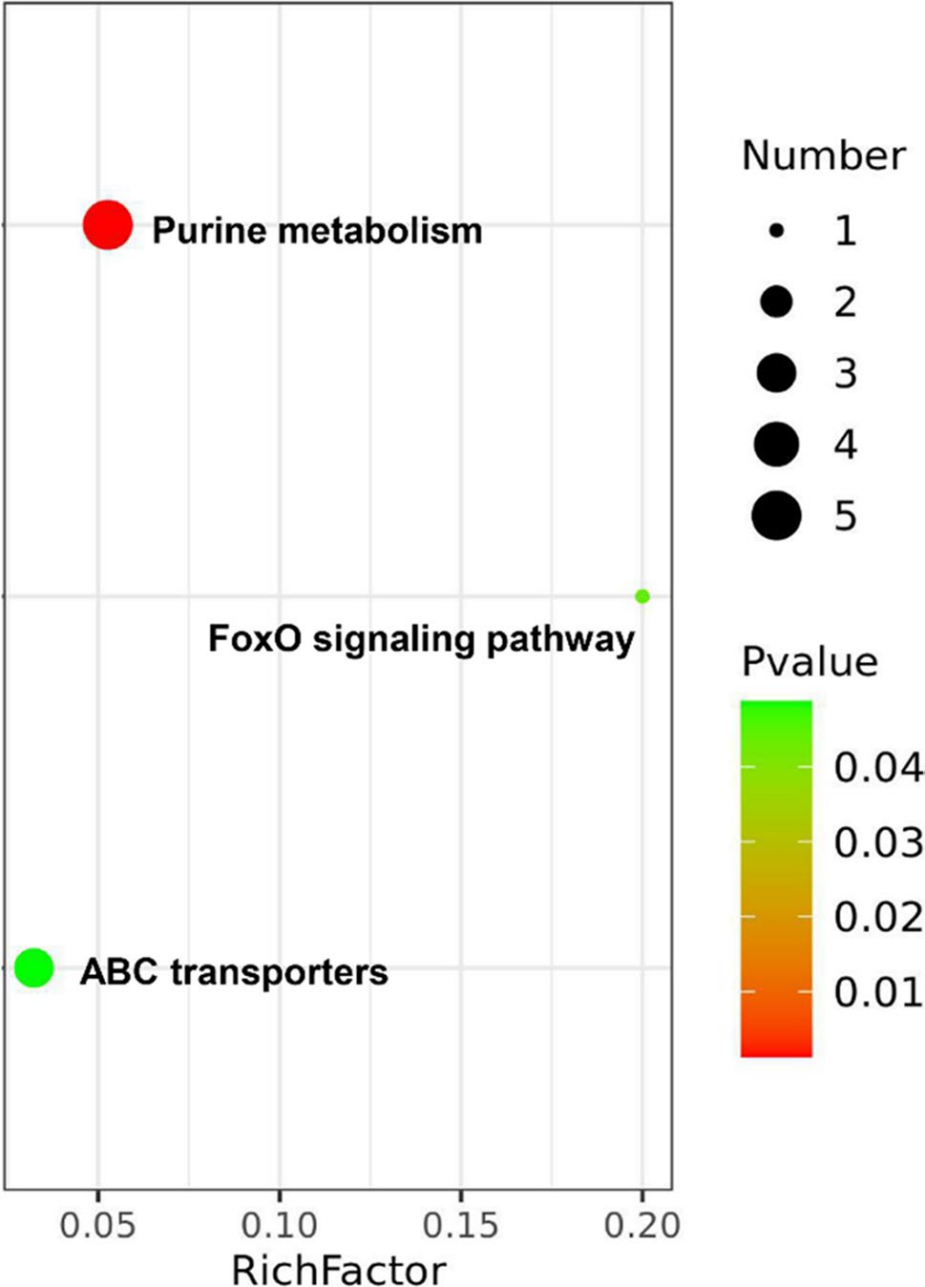
Metabonomics pathway analysis. Based on the impact value > 0.05, two perturbed metabolic pathways, including purine metabolism and FoxO signaling pathway were significantly affected by Sal B

In the score plot, scattered points of various samples were segregated into three groups, suggesting different metabolic patterns with Ang II treatment or Sal B + Ang II treatment. One hundred twenty-one potential Ang II–related metabolites with a VIP value of > 1.0 and a significance test *p* < 0.05 were displayed on the loading plot. The databases HMDB (http://www.hmdb.cn), ChemSpider (http://www.chemspider.com), Lipidmaps (http://www.lipidmaps.org), and KEGG (http://www.kegg.com) were used to search the candidate metabolites using masses and MSE data. According to the results of the screening of different metabolites and the relative abundance of the metabolites, comparing the Ang II group with the control group, there were 23 different metabolites with important endogenous differences. After being pre-treated with Sal B, the levels of the above metabolites were partially reversed to normal (Table 2). We carried out a systematic pathway analysis of the metabonomics to explore the biomarkers. MetaboAnalyst 4.0 (http://www.metaboanalyst.ca/), an online software, was used for pathway enrichment analysis. Based on the impact value > 0.05, two perturbed metabolic pathways, including purine metabolism and FoxO signaling pathway, were significantly affected by Sal B (Fig. 4).

**Table 2:** Metabolites as biomarkers characterized in cell profile and their change trends (*n* = 6 per group)

Metabolites	VIP	*p*	Ang II^a^	Ang II + Sal B^b^
(R)-2-Ethylmalic acid	1.25	0.001019759	↓(*)	↑(*)
3-Oxo-OPC4-CoA	1.11	0.03388	↑(*)	↓
ADP-ribose	1.09	0.003818	↑(*)	↓
Adenosine monophosphate (AMP)	1.40	0.038411998	↓(*)	↑(*)
Guanosine	2.19	0.03735	↑(*)	↓(*)
d-Galactose	5.83	0.000246954	↑(*)	↓(*)
d-Fructose	5.01	3.59721E-06	↑(*)	↓(*)
d-Glucose	8.38	0.003175685	↑(*)	↓(*)
Adenine	2.12	0.003404153	↑	↓(*)
l-Glutamate	4.68	0.020588355	↓(*)	↑(*)
Fumaric acid	1.01	0.003105849	↓(*)	↑(*)
Raffinose	1.15	0.001459291	↓(*)	↑(*)
Uridine 5′-monophosphate (UMP)	1.86	0.000750348	↑ (*)	↓(*)
Biliverdin	3.81	0.002138302	↓(*)	↑(*)
Biotinyl-5′-AMP	1.01	0.000213036	↑(*)	↓(*)
Butyryl-CoA	3.55	1.04537E-07	↑(*)	↓(*)
Cholic acid	4.27	9.12687E-05	↑(*)	↓(*)
Dimethylbenzimidazole	1.95	1.32008E-06	↑(*)	↓(*)
O-Phosphohomoserine	1.22	0.000100527	↓(*)	↑(*)
Pantothenic Acid	3.12	0.046269276	↓(*)	↑(*)
Phenylacetylglycine	4.61	3.58928E-06	↑(*)	↓
Phosphocreatine (PCr)	3.19	0.037098375	↓	↑(*)
Phosphoribosyl-AMP	1.3202	0.0059	↑(*)	↓

^a^Change trend compared with control group

^b^Change trend compared with Ang II group

The levels of potential biomarkers were labeled with (↓) downregulated and (↑) upregulated (*p < 0.05)

### Sal B Induced AMPK/FoxO1 Activation and miR-148a-3p Expression in Ang II–Activated MFBs

**Fig. 5:**
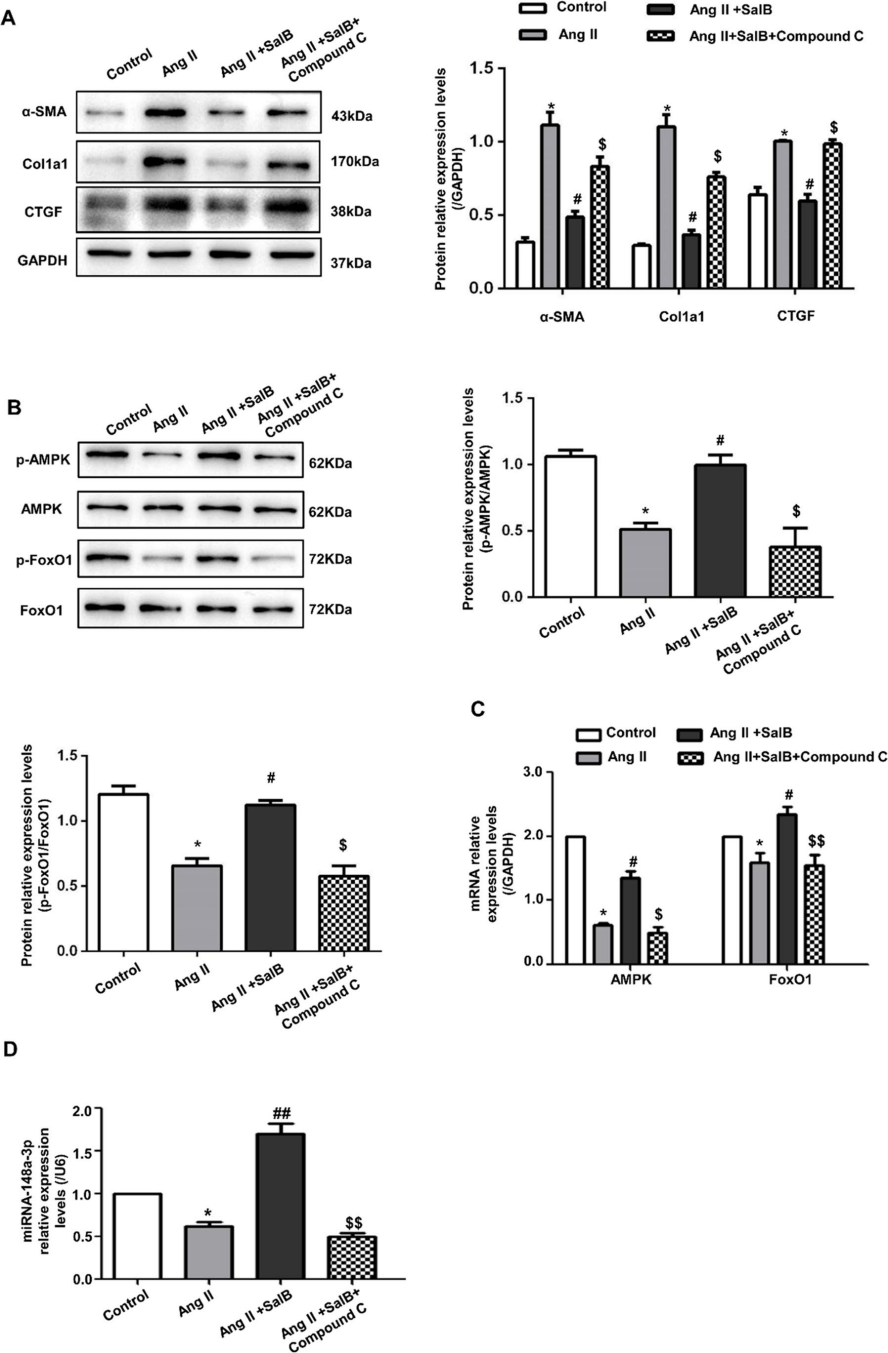
Sal B induced AMPK/FoxO1 activation and miR-148a-3p expression in Ang II–activated MFBs. **A** Western blot showed that the protein expressions of α-SMA, Col1a1, and CTGF in control, Ang II, Ang II + Sal B, and Sal B + Compound C (AMPK antagonist) groups. **B** Western blot showed that the phosphorylation of AMPK and FoxO1 in each group. **C** RT-PCR analysis of AMPK and FoxO1 levels in each group. Data were normalized to GAPDH. **D** The levels of miR-148a-3p in untreated FBs (control), Ang II–treated FBs, and Ang II + Sal B–treated FBs and Sal B + Compound C–treated FBs after 24 h. Data were normalized to U6. Data were presented as mean ± SEM (*n* = 3 in each group). *p < 0.05 vs control group, **p < 0.01 vs control group; #*p* < 0.05 vs Ang II group, ##*p* < 0.01 vs Ang II group; $*p* < 0.05 vs Ang II + Sal B group, $$*p* < 0.01 vs Sal B + Compound C group

FoxO1, a member of the forkhead box transcription factor family, has been shown to inhibit the activation of fibroblasts and the production of ECM, and have an anti-fibrotic effect on organs such as the heart. FoxO signaling pathway may also protect the cardiac muscles in the early stage of heart diseases [28]. To explore the exact mechanism of Sal B causing the changes of metabolites by FoxO signaling pathway, we also introduced AMP-activated protein kinase (AMPK), an upstream modulator of FoxO1 activity [29], could inhibit the progression of liver fibrosis by activating its downstream targets such as FoxO1 [30]. To study the key role of AMPK in the protective effect of Sal B against atrial fibrosis, FBs stimulated with Ang II were co-incubated with Sal B and AMPK inhibitor Compound C. Western blot analysis demonstrated that the protein expressions of α-SMA, Col1a1, and CTGF were significantly upregulated in Ang II–activated FBs after Sal B and Compound C pre-treatment when compared to Ang II + Sal B group (Fig. 5A). Corresponding with the upregulation of fibrosis-related protein expressions, p-AMPK and p-FoxO1 were reduced in the Sal B + Compound C group (Fig. 5B). The phosphorylation of AMPK and FoxO1 induced by Sal B was inhibited and further blocks the anti-fibrotic effect of Sal B after being pre-treated by Compound C. This suggested that the anti-fibrotic effect of Sal B was partly mediated by AMPK/FoxO1. Consistent with this, in qRT-PCR, when the cells were co-incubated with Ang II and Sal B, the phosphorylation of AMPK and FoxO1 was significantly increased. The above changes were partially reversed when treated with Compound C (Fig. 5C).

MiR-148a-3p, a member of miR-148/-152 family, has been shown to be involved in the pathogenesis of fibrosis in previous studies [31]. FoxO1 was a transcription factor for miR-148a-3p gene expression [32], and the regulation of miRNA expression during oxidative stress by FoxO1 is important for the normal function of cardiomyocytes [33]. We studied the role of AMPK/FoxO1 activation and miR-148a-3p expression in the anti-fibrosis effect of Sal B. Specifically, we investigated the expression of AMPK/FoxO1/miR-148a-3p axis in Ang II–activated FBs. qRT-PCR assay showed that compared with the control group, Ang II inhibited the expression of miR-148a-3p. When cells were co-incubated by Ang II and Sal B, the expression level of miR-148a-3p was significantly higher than that in the Ang II groups (*p* < 0.05) (Fig. 5D). The high expression of miR-148a-3p induced by Sal B was inhibited after being pre-treated with Compound C.

**Fig. 6:**
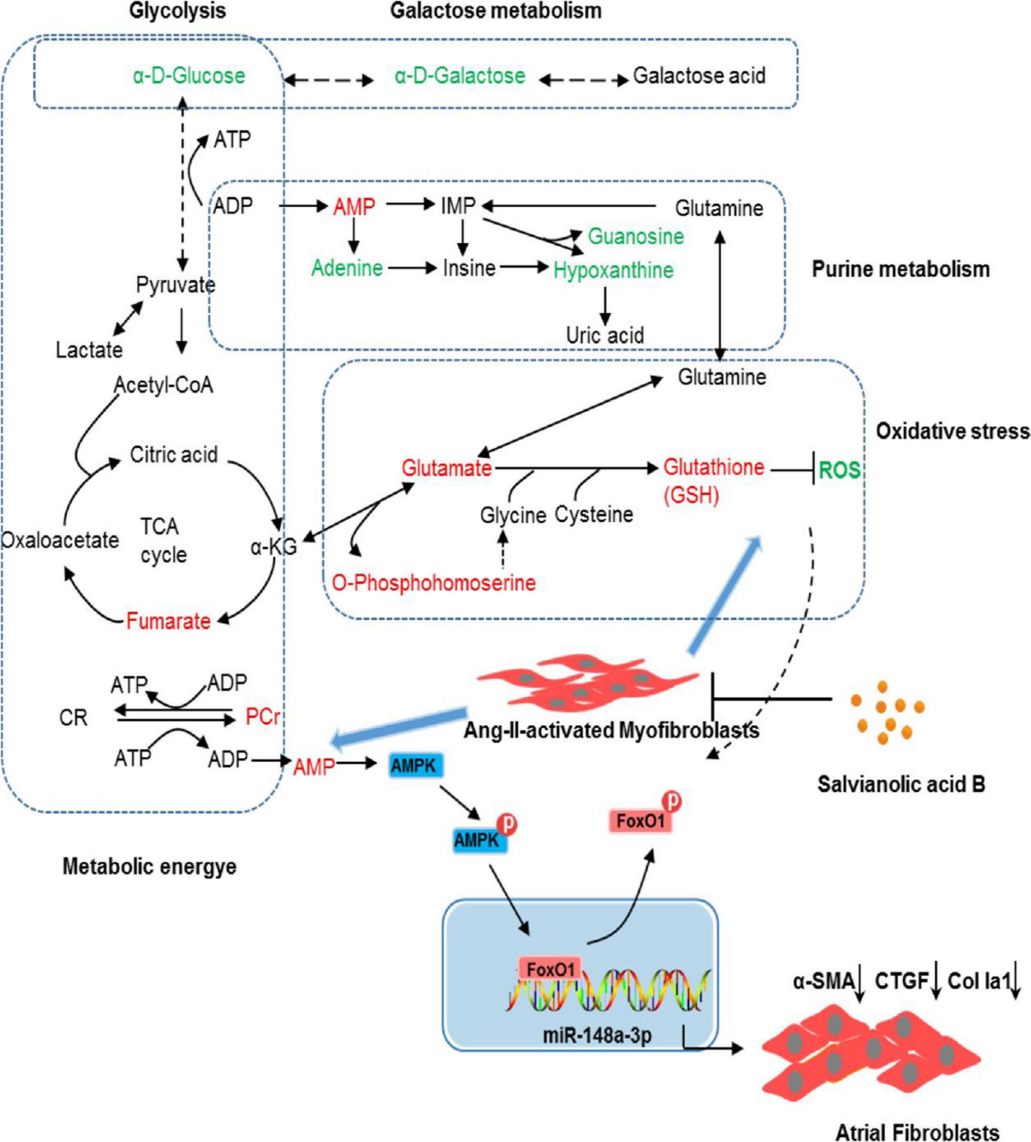
Schematic diagram of the perturbed metabolic pathways regulated by Sal B for the beneficial effects in Ang II–activated cardiac fibroblasts. Green and red represent reduction and increase in the Sal B–treated group compared with the Ang II group, respectively

Collectively, these results showed that Sal B can dramatically reduce the stimulating effects of Ang II on the accumulation of ECM via miR-148a-3p induction by activating AMPK/FoxO1 pathway. A schematic diagram of perturbed metabolism was constructed based on the relationship between the biochemical factors and differential metabolites (Fig. 6).

## Discussion

It has been shown that Sal B attenuates cardiac fibrotic remodeling by reducing the Ang II–induced proliferation and differentiation of FBs into MFBs [10]. However, the atrial anti-fibrotic mechanism of Sal B remains unclear. This research firstly explored the mechanism underlying Sal B activity in Ang II–stimulated FBs via an untargeted metabolomic analysis. Sal B altered the metabolic pattern of Ang II–activated FBs, and significantly inhibited the cell proliferation and secretion of ECM proteins. Biochemical indicators and metabolic profile analysis including PCA, PLS-DA, and OPLS-DA score plots also supported the anti-fibrotic activity of Sal B. Metabolomic analysis showed that a recovery impact of Sal B on Ang II–activated FBs, including purine metabolism and FoxO signaling pathway, associated with energy metabolism and oxidative stress. We further verified that the protective effect of Sal B on atrial fibrosis was partly medicated by miR-148a-3p via AMPK and FoxO1 activation (Fig. 6).

Atrial fibrosis is the most significant feature of atrial remodeling in patients with AF, which can lead to the atrial abnormalities in electric conduction and excitement, providing an important pathophysiological basis for the recurrences, resistance to therapy, and complications of AF [2]. The trans-differentiation of FBs into MFBs is a key event in the complex process of AF, involving a variety of ECM proteins secreted by Ang II–activated FBs [34]. Therefore, control of the abnormal proliferation of FBs and differentiation into MFBs is crucial to attenuate atrial fibrosis.

Ang II, the major mediator of the renin-angiotensin system, contributes to the pathogenesis of diverse organic fibrosis. Ang II could promote the proliferation, migration, differentiation, and ECM synthesis of FBs. So, in this study, we investigated the effects of Sal B on Ang II–induced cellular functional properties of FBs. Immunofluorescence staining of α-SMA confirmed that Ang II induced a significant upregulation of α-SMA expression in FBs. We also measured the inhibiting effect of Sal B based on the expression of fibrosis-related mRNA and protein expressions. Compared with the Ang II group, Sal B pretreatment could partly negate Ang II–induced decrease of intracellular GSH level, alleviate Ang II–induced oxidative stress of FBs, significantly downregulate the protein expressions of Col1a1 and CTGF, and reduce the ECM synthesis of FBs and its differentiation into MFBs.

MFBs can be activated by a variety of signals that serve as markers of cellular injury, including pro-inflammatory cytokines produced by infiltrating immune cells, hepatocyte apoptotic bodies, endothelial cell–mediated growth factor activation, and ROS [35]. The cellular response to injury is potentiated by a series of paracrine and autocrine loops, including fibrogenic signals such as TGF-β1 and CTGF. Activated MFBs have increased energy requirement to support cell proliferation; production of ECM proteins, proteases, and cytokines; and migration [10]. A number of metabolic pathways were harnessed to meet these energy demands. This is in some ways similar to cancer cells [36]. Our metabolomic analysis revealed that compared with the control group, intracellular phosphocreatine (PCr), a compound known to serve as an intracellular energy reserve, decreased in Ang II–stimulated FBs. Meanwhile, fumaric acid, as an intermediate product of the tricarboxylic acid cycle (TCA), decreased in FBs after Ang II stimulation, indicating that Ang II stimulation inhibited the TCA cycle [37]. Moreover, Ang II increased intracellular glucose levels which were involved in the glycolysis pathway [35]. These results indicated that Ang II induced the abnormal energy metabolism to meet ATP production and insufficient energy supply [38]. These results were consistent with previous studies [39] which showed that activated FBs required a large amount of energy to support cell proliferation as well as the secretion of ECM, protease, and cytokines through PCr transformation [40] and fatty acid oxidation [41]. After pre-treatment with Sal B, the above substances were partly restored to normal, while the content of AMP was also increased. Previous studies revealed that increased AMP or AMP/ATP ratio could directly activate AMPK by binding to the AMPK-γ regulatory subunit [42]. As a central energy sensor, AMPK was involved in myocardial fibrosis [43]. Recently, experiments showed that AMPK could inhibit the progression of fibrosis by activating its downstream targets such as FoxO1 [44, 45].

The changes in the content of AMP, PCr, GSH, and ROS in FBs lead us to postulate that Sal B exerts its anti-fibrotic effect by activating AMPK/FoxO1. In our study, metabolic network analysis showed that AMP was involved in FoxO signaling pathway in Ang II–induced myocardial fibrosis. Sal B not only modulated the abnormal metabolism but also profoundly elevated the excessive AMP to active AMPK and downstream FoxO1. We further verified the above findings in the *in vitro* experiments (Fig. 5). Ang II inhibited phosphorylation of AMPK and FoxO1 and inhibited expression of miR-148a-3p in activated FBs. When cells were co-incubated by Ang II and Sal B, the phosphorylation of AMPK was significantly activated, which corresponded to miR-148a-3p expression mediated by FoxO1 activation. In addition, the anti-fibrotic effect caused by Sal B was blocked after being pre-treated with Sal B and Compound C in Ang II–activated FBs. These findings provided direct evidence that Sal B can reverse the pro-fibrotic effect of Ang II on FBs via the AMPK/FoxO1/miR-148a-3p pathway. Meanwhile, PCr, AMP, and glutamate (Glu) can be potentially to use as biomarkers for monitoring the anti-fibrotic effect of Sal B.

Moreover, metabolic network analysis showed that Glu was identified as one of the differential metabolites and was also involved in FoxO signaling pathway induced by Ang II. In this study, the alteration of GSH and Glu reflected the antioxidant role of Sal B against Ang II–triggered ROS production in FBs. The levels of intracellular Glu and GSH were decreased in Ang II–induced FBs, indicating the synthesis of GSH was blocked. O-Phosphoserine, as a substrate for serine, is also involved in the glycine synthesis [46], which is essential for GSH. Ang II decreased the level of O-phosphoserine in cells which partly blocked the synthesis of GSH. After intervention, Sal B promoted the synthesis of GSH and decreased the ROS production in Ang II–induced FBs. The production or release of nitric oxide (NO) and ROS can lead to severe oxidative damage [47]. Therefore, Sal B could elevate the intracellular GSH level and potentially abolished Ang II–induced oxidative stress. In addition, Sal B could significantly downregulate the expression of Col1a1 and CTGF proteins, reducing the production of ECM by myofibroblasts. These results suggested that Sal B may be involved in antioxidant stress and anti-fibrotic effect by activating AMPK/FoxO1 pathway.

In our study, purine metabolism is also involved in Ang II–induced atrial fibrosis through pathway enrichment analysis. Purines occur in all organisms as the bases of DNA and RNA (such as adenine, guanine) and of nucleotides (ATP and GTP), playing an important role in energy transfer and metabolic regulation [46]. Similar to the activation of hepatocytes, a large amount of energy and nucleotides was necessary for the abnormal proliferation of FBs and the secretion of ECM. Besides, metabolic reprogramming would be activated by the activation of hepatic stellate cell and subsequent secretion of ECM. In this study, guanosine, hypoxanthine, and adenine were the main substances involved in purine metabolism, not only participating in the synthesis of DNA and RNA, but also providing necessary energy. Their abnormal increase in Ang II–activated FBs may be due to the increased demand for energy and nucleic acids for cell proliferation and ECM production. Increased purine metabolism may result in the accumulation of xanthine [48]. Xanthine oxidase, the last enzyme involved in purine metabolism, catalyzes the oxidation of hypoxanthine to xanthine and produces superoxide radicals [49]. The study found that oxygen free radicals can reduce the content of ATPase and somatostatin in cells and affect the proliferation and differentiation of cells, leading to the destruction of cell structure [50]. However, after Sal B treatment, their levels were significantly downregulated, mainly because the intracellular guanine nucleoside level was reversed to normal.

## Conclusion

Combining pharmacodynamics with UPLC-Q/TOF–MS analysis, metabolomics approach was used to evaluate the protective effects of Sal B on atrial fibrosis on the cellular level. The identified metabolites were related to the two metabolic pathways including FoxO signaling pathway and purine metabolism. Sal B could ameliorate atrial fibrosis through miR-148a-3p induction by activating the AMPK/FoxO1 pathway. This enhanced mechanistic understanding of Sal B activity could further promote the development of Sal B as a potential therapeutic intervention for artial fibrosis.

## Data Availability

All the data generated or analyzed during this study are included in this published article.

## Abbreviations

AF: Atrial fibrillation
Ang II: Angiotensin II
AMPK: AMP-activated protein kinase
α-SMA: Alpha smooth actin
BCA: Bicinchoninic acid
CTGF: Connective tissue growth factor
Col1a1: Collagen I
Col3a1: Collagen III
CCK8: Cell counting kit 8
cDNA: Complementary DNA
DMEM: Dulbecco’s modified eagle’s medium
DCFDA: Dichlorofluorescin diacetate
DAPI: 4,6-Diamidino-2-phenylindole
ECM: Extracellular matrix
EdU: 5-Ethynyl-20-deoxyuridine
FBS: Fetal calf serum
FBs: Fibroblasts
GSH: Glutathione
Glu: Glutamate
HSC: Hepatic stellate cell
H&E: Hematoxylin and eosin
MFBs: Myofibroblasts
MCAO: Middle cerebral artery occlusion
NO: Nitric oxide
OPLS-DA: Orthogonal partial least squares-discriminate analysis
PCr: Phosphocreatine
PCA: Principle component analysis
PLS-DA: Partial least squares-discriminate analysis
ROS: Reactive oxygen species
Sal B: Salvianolic acid B
TCA: Tricarboxylic acid cycle
UPLC-Q-TOF-MS: Ultra-performance liquid chromatography/quadrupole time-of-flight mass spectrometry
